# An analysis of factors influencing pulmonary artery catheter passage through the tricuspid and pulmonary valves

**DOI:** 10.1186/s40981-020-00344-5

**Published:** 2020-05-23

**Authors:** Yuka Miyata, Shoko Takada, Tomoko Fujimoto, Mitsuo Iwasaki, Yukio Hayashi

**Affiliations:** 1 Anesthesiology Service, Sakurabashi-Watanabe Hospital, 2-4-32 Umeda, Kita-ku, Osaka, 530-0001 Japan; 2 Department of Anesthesiology, Osaka University Hospital, 2-2 Yamada-oka, Suita, Osaka, 565-0871 Japan

**Keywords:** Pulmonary artery catheter, Difficult placement, Tricuspid valve, Pulmonary valve, Aortic root

## Abstract

**Purpose:**

A pulmonary artery catheter (PAC) has to pass the tricuspid and pulmonary valves for its proper placement. Although several factors were reported to hinder the placement, there have been no reports to identify the factors that prolong the individual time for passing through each valve.

**Method:**

We individually measured the time required for a PAC to pass through the tricuspid and pulmonary valves. We examined the effect of the following factors on those times: the patient’s age, sex, height, weight, cardiothoracic ratio, tricuspid regurgitation, left ventricular ejection fraction, and the diameters of the sinus of Valsalva and of the sinotubular junction divided by the body surface area which represent the diameter of the aorta. Data were analyzed by multiple linear regression analysis after univariate analysis.

**Results:**

The placement of a PAC was successful in all of 100 patients. The time required to pass through the pulmonary valve was significantly longer than that through the tricuspid valve (15 [10–28] s vs 9 [5–16] s, median [range], *P* < 0.01). The incidence of ventricular arrhythmias during passage through the pulmonary valve was significantly higher than that through the tricuspid valve (17% vs 0%, *P* < 0.01). Tricuspid regurgitation and the diameter of sinotubular junction had a significant positive association with the time required to advance a PAC through the pulmonary valve, although there was no significant factors that increased the time required to advance a PAC through the tricuspid valve.

**Conclusion:**

The time required to advance a PAC through the pulmonary valve is much longer than that to pass through the tricuspid valve. The diameter of aortic root and tricuspid regurgitation are significant factors that increased the time required to advance a PAC through the pulmonary valve.

## Introduction

Pulmonary artery catheter (PAC) placement is a common procedure in anesthetic management of patients undergoing cardiovascular surgery, although the application of the catheter is still remained controversial [1, 2]. In our hospital, the catheter is routinely placed after induction of anesthesia by observing the pressure waves. The catheter with a balloon on its tip carried by the blood stream has to pass the two valves, that is, tricuspid and pulmonary valves, to reach the pulmonary artery. So far, several reports have examined the factors that prolong time to place a PAC and several patient-related factors have been reported [3–8]. However, there have been no reports to identify the factors to longer time to pass through tricuspid and pulmonary valves, respectively. Thus, the present study was designed as an extension of our previous work [6–8] to examine the time required for the catheter passage through the each valve in adult patients undergoing cardiovascular surgery and we examined factors to longer time to pass through tricuspid and pulmonary valves, respectively.

## Methods

The current study was approved by the institutional review board, and informed consent was obtained from all eligible patients and was registered in the UMIN Clinical Trial Registry (UMIN 000027418). This study was conducted from October 2017 to December 2018 at Sakurabashi-Watanabe Hospital in Osaka, Japan. We prospectively examined the time required for the PAC placement for 100 adult patients undergoing elective cardiovascular surgery. The patients who had a history of tricuspid ring annuloplasty were excluded [8]. All patients were monitored electrocardiogram, invasive arterial pressure, oxygen saturation, and end-tidal carbon dioxide. After the induction of anesthesia with midazolam 5 mg, fentanyl 0.2 mg, and vecuronium 8 mg, mechanical ventilation was started following tracheal intubation. Anesthesia was maintained with propofol or sevoflurane combined with remifentanil and fentanyl. The PAC (continuous cardiac output/SvO_2_ Catheter 744HF75, Edwards Lifesciences, Irvine, CA, USA) was inserted through the right internal jugular vein. The placement of PAC was performed by experienced physicians who are certified anesthesiologists of the Japan Society of Anesthesiologists. First, the introducer sheath was placed via the right internal jugular vein in the Trendelenburg position, and then, the PAC was started floating through the sheath by monitoring the pressure waveform in the flat position. The PAC was inserted approximately 20 cm and central venous pressure waveform was confirmed; subsequently, the balloon was inflated with 1.5 ml of air. With inflated balloon, the tip of the catheter was floated into the right ventricle, and then, it was placed in the pulmonary artery. The waveform of the pulmonary artery was first observed, followed by inserting the catheter approximately 2–3 cm forward and deflated the balloon. In this position, being confirmed that the tip of the catheter was not wedged into the pulmonary artery, the catheter was locked with the sheath.

The time required for a PAC to pass through the tricuspid and pulmonary valve was separately measured. The catheter passage time for the tricuspid valve and the pulmonary valve was defined as the time from the CVP position to the right ventricle through the tricuspid valve and that from the right ventricle to the pulmonary artery through the pulmonary valve, respectively. That is, the first beginning time point was just after the inflation of the balloon to start floating the catheter and the first ending time point is the time which we first observed the waveform of the right ventricle. After confirmation of the waveform of the right ventricle, we restated floating the catheter. The second begging time was to restart floating the catheter with the waveform of the right ventricle and the second ending time point is the time which we first observed the waveform of the pulmonary artery. If the placement failed to precede the catheter into the pulmonary artery within 5 min, some guidance such as transesophageal echocardiography or X-ray fluoroscopic system to visualize intracardiac catheter orientation was used.

In this study, we examined the effect of the following factors, which covered the patient’s characteristics, cardiac size and aortic size and cardiac function, on each catheter passage time; the patient’s age, sex, height, weight, body mass index (BMI), cardiothoracic ratio (CTR), left ventricular ejection fraction (LVEF), the degree of tricuspid valve regurgitation (TR), atrial fibrillation, the diameter of the sinus of Valsalva (S Val)/body surface area (BSA), and the diameter of the sinotubular junction (STJ)/body surface area (BSA) which represent the diameter of the aorta. LVEF and the degree of TR and CTR were evaluated by transthoracic echocardiography and X-ray examination, respectively, prior to the surgery. The diameter of S Val and STJ was obtained by the transesophageal echocardiography in the mid-esophageal long axis view after the induction of anesthesia to evaluate the effect of the aortic diameter on the PAC placement. This measurement was done by a staff physician who was blind to the catheter placement time and was done at midsystole from inner edge to inner edge, because the catheter was floated through the pulmonary valve during systole. According to the guideline [9], we recorded this value divided by BSA to equalize among different physical sizes. In addition, we recorded electrocardiography tracing continuously during the PAC placement in our monitoring system for review arrhythmias. We defined ventricular arrhythmias as three or more consecutive ventricular arrhythmias in this study [10].

### Sample size calculation and statistics

Based on our previous study [6], we collected 100 cases, and then, if needed more cases, we planned to calculate sample size. Actually, we found statistically significant factors with these 100 cases. Thus, we decided to report with this sample size.

Data were expressed as means ± SD or as a median range and interquartile range as appropriate. The time duration through the tricuspid and that through the pulmonary valves was compared by Mann-Whitney, and occurrence of the ventricular arrhythmias was analyzed by Fisher’s exact test. To predict the difficulty of PAC passage through each valve from the factors, a multiple linear regression was conducted. Factors included in multiple linear regression analysis were selected among variables yielding *P* < 0.2 by simple linear regression analysis. All analyses were conducted with SPSS (IBM Corporation, USA) version 20.0. *P* < 0.05 was considered statically significant.

## Results

The placement of a PAC was successful in all of 100 patients. The patient’s demographic data including patient’s disease targeted to surgery is presented in Table 1. The time duration to advance the catheter through the tricuspid and pulmonary valves and incidence of ventricular arrhythmias during passage of each valve is shown in Table 2. The time duration through the pulmonary valve was significantly longer than that through the tricuspid valve, and incidence of ventricular arrhythmias was also significantly higher. The results of simple and multiple linear regression models of potential predictors of increased time duration to advance the catheter through the tricuspid valve and the pulmonary valve are shown in Tables 3 and 4, respectively. There were no significant factors to increase the time duration to advance through the tricuspid valve (Table 3), whereas TR and STJ/BSA had a significant positive association with the time duration to advance the catheter through the pulmonary valve (Table 4). Here, the two aortic root factors (sinus of Valsalva and STJ) significantly may correlate with each other, so we chose the diameter of STJ/BSA which represented the diameter of the aortic root for the following multivariate analysis because of higher a *P* value in the univariate analysis.

**Table 1:** Summary of 100 patients

Age (year)	67 ± 13
Sex (male/female)	73/27
Height (cm)	164 ± 12
Weight (kg)	63 ± 13
CTR (%)	52 ± 7
LVEF (%)	62 ± 14
Degree of TR	1 (0–1)
STJ/BSA (mm/m^2^)	15.6 ± 2.8
Disease	AS (20) AR (12) AS + AR (2) MR (17) TR (1) CAD (11) DCM (2) TAA (22) AAE (4) CAD + AS (2) CAD + MR (1) AR + MR (4) AS + MS (1) Myxoma (1)

Data were expressed as means ± SD except for the degree of tricuspid regurgitation, which is expressed as median (interquartile range)

*CTR* cardiothoracic ratio, *LVEF* left ventricular ejection fraction, *TR* tricuspid regurgitation, *STJ* sinotubular junction, *AS* aortic stenosis, *AR* aortic regurgitation, *MR* mitral regurgitation, *TR* tricuspid regurgitation, *CAD* coronary artery disease, *DCM* dilated cardiomyopathy, *TAA* thoracic aorta aneurysm, *AAE* annuloaortic ectasia

**Table 2:** Time required to advance the pulmonary artery catheter through the tricuspid and pulmonary valves and incidence of ventricular arrhythmias during passage of each valve

	Tricuspid valve	Pulmonary valve	*P* value
Time duration (s)	9 (5–16)	15 (10–28)	< 0.001
Ventricular arrhythmias *n* (%)	0 (0)	17 (17)	*<* 0.001

Data (time duration) were expressed as median (interquartile range)

**Table 3:** Simple (A) and multiple (B) linear regression model of potential predictors of increased time duration to advance the catheter through the tricuspid valve

	Parameter estimation (95% confidence limits)	SE	*P* value
A			
Age (year)	0.371 (− 0.197–0.938)	0.296	0.198
Sex (male)	− 10.630 (− 27.029–5.769)	8.264	0.201
Height (cm)	− 0.287 (− 0.897–0.324)	0.308	0.354
Weight (kg)	− 0.087 (− 0.642–0.463)	0.280	0.755
BMI (kg/m^2^)	0.633 (− 1.435–2.701)	1.042	0.545
CTR (%)	0.469 (− 0.610–1.548)	0.544	0.391
LVEF (%)	0.104 (− 0.442–0.649)	0.275	0.706
Degree of TR	6.993 (− 0.578–14.564)	3.815	0.070
Atrial fibrillation	− 8.441 (− 31.844–14.962)	11.793	0.476
S Val/BSA (mm/m^2^)	− 0.961 (− 3.202–1.280)	1.129	0.397
STJ/BSA (mm/m^2^)	− 1.696 (− 4.331–0.939)	1.328	0.205
B			
Age (year)	0.263 (− 0.316–0.842)	0.292	0.370
Degree of TR	6.160 (− 1.638–13.959)	3.929	0.120

*BMI* body mass index, *CTR* cardiothoracic ratio, *LVEF* left ventricular ejection fraction, *TR* tricuspid regurgitation, *S Val* sinus of Valsalva, *STJ* sinotubular junction

**Table 4:** Simple (A) and multiple (B) linear regression model of potential predictors of increased time duration to advance the catheter through the pulmonary valve

	Parameter estimation (95% confidence limits)	SE	*P* value
A			
Age (year)	0.062 (− 0.270–0.394)	0.167	0.712
Sex (male)	4.497 (− 5.076–14.070)	4.824	0.354
Height (cm)	0.167 (− 0.188–0.522)	0.179	0.353
Weight (kg)	− 0.038 (− 0.361–0.285)	0.163	0.815
BMI (kg/m^2^)	− 0.747 (− 1.942–0.448)	0.602	0.218
CTR (%)	0.669 (0.053–1.284)	0.310	0.034
LVEF (%)	0.012 (− 0.306–0.329)	0.160	0.941
Degree of TR	7.063 (2.815–11.310)	2.140	0.001
Atrial fibrillation	23.842 (11.063–36.621)	6.44	0.001
S Val/BSA (mm/m^2^)	2.232 (0.896–3.368)	0.623	0.001
STJ/BSA (mm/m^2^)	3.383 (1.995–4771)	0.699	< 0.001
B			
CTR (%)	− 0.087 (− 0.708–0.535)	0.313	0.783
Degree of TR	4.664 (0.070–9.258)	2.314	0.047
Atrial fibrillation	8.380 (− 6.603–23.362)	7.547	0.270
STJ/BSA (mm/m^2^)	2.897 (1.422–4.371)	0.743	0.001

*BMI* body mass index, *CTR* cardiothoracic ratio, *LVEF* left ventricular ejection fraction, *TR* tricuspid regurgitation, *S Val* sinus of Valsalva, *STJ* sinotubular junction

## Discussion

The present study showed that the time required to advance the PAC through the pulmonary valve is much longer than that to pass through the tricuspid valve and ventricular arrhythmias during PAC placement predominantly occur during passage of the pulmonary valve (Table 2). We also found that the diameter of aortic root and TR are significant factors to increase the time duration to advance the PAC through the pulmonary valve, although we could not find any factor to prolong to pass the tricuspid valve (Tables 3 and 4).

Although previous clinical researches and case reports have shown several factors to be associated with difficult PAC placement [3–8], there are no reports to examine the time required to individually advance a PAC through the tricuspid and the pulmonary valves. The present study is the first report to show risk factors to prolong passage time through each valve, respectively, and we clarified that STJ/BSA was a significant factor to prolong passage of the pulmonary valve, not the tricuspid valve (Tables 3 and 4). The ascending aorta and pulmonary artery are anatomically enclosed by the pericardium, and the pulmonary valve is located slightly upper and anterior to the aortic valve. Dilation of the aortic root would shift the right ventricular outflow tract and/or the ostial pulmonary artery, where the PAC passes. Thus, the shift of anatomical change of this route would be time-consuming for the PAC placement.

In this study, we also identified TR as another factor to prolong the passage through the pulmonary valve (Table 4), although our previous study could not show that TR was a significant factor to longer time to place a PAC [6]. We supposed that the present result may be reasonable. Blood flow from the right ventricle to the pulmonary artery during the systolic period may play a significant role to advance the PAC to pass through the pulmonary valve. However, the presence of TR would produce turbulence of the blood flow in the right ventricle, resulting in attenuating the driving force to advance the PAC to the pulmonary artery.

In contrast with the pulmonary valve, we could not find any risk factor to prolong the passage through the tricuspid valve by the multiple variant analyses (Table 3). Considering that the number of the subjects in this study was 100, we have to acknowledge the possibility that we could have found some factors to prolong passage through the tricuspid valve with increasing the number of observation.

The experience is well known to be an important factor for the PAC placement [5, 11]. We designed the present study to include the data by staff anesthesiologists to eliminate the effect of experience of anesthesiologist. Nevertheless, technical individual differences between anesthesiologists may affect the present results, even if they are experienced anesthesiologists. Thus, further study may be needed to elucidate the effect of experience and technical individual differences on PAC placement.

We have to discuss potential limitations in our study. First, we chose the eleven variables in this study, so we have to acknowledge the possibility that we overlook another important factor to affect the time durations we measured. Thus, if we had missed an important factor, our results would have to be reexamined. Second, our results may reach the statistical significance about the diameter of sinotubular junction/BSA (Table 4). However, the results are dependent on the statistical analysis and the clinical significance of our results would be interpreted with caution.

## Conclusions

In conclusion, the present study showed that the time duration to advance the PAC through the pulmonary valve is much longer than that to pass through the tricuspid valve. We also found that the diameter of the aortic root and TR are significant factors to increase the time duration to advance the PAC through the pulmonary valve.

## Data Availability

The data that support the findings of this study are available from the corresponding author on reasonable reason.
